# Recent Progress of Electrochemical Aptasensors toward AFB1 Detection (2018–2023)

**DOI:** 10.3390/bios14010007

**Published:** 2023-12-22

**Authors:** Despina Ciobanu, Oana Hosu-Stancioiu, Gheorghe Melinte, Flavia Ognean, Ioan Simon, Cecilia Cristea

**Affiliations:** 1 Department of Analytical Chemistry, Faculty of Pharmacy, “Iuliu Haţieganu” University of Medicine and Pharmacy, 4 Pasteur Street, 400349 Cluj-Napoca, Romania; ioana.desp.ciobanu@elearn.umfcluj.ro (D.C.); melinte.gheorghe@umfcluj.ro (G.M.); flavia.anam.ognean@elearn.umfcluj.ro (F.O.); 2 Department of Surgery, Faculty of Medicine, “Iuliu Haţieganu” University of Medicine and Pharmacy, 400012 Cluj-Napoca, Romania; ioan.simon@umfcluj.ro

**Keywords:** aflatoxin B1, aptamer, electrochemical, labeled, label-free aptasensors

## Abstract

Food contaminants represent possible threats to humans and animals as severe food safety hazards. Prolonged exposure to contaminated food often leads to chronic diseases such as cancer, kidney or liver failure, immunosuppression, or genotoxicity. Aflatoxins are naturally produced by strains of the fungi species *Aspergillus*, which is one of the most critical and poisonous food contaminants worldwide. Given the high percentage of contaminated food products, traditional detection methods often prove inadequate. Thus, it becomes imperative to develop fast, accurate, and easy-to-use analytical methods to enable safe food products and good practices policies. Focusing on the recent progress (2018–2023) of electrochemical aptasensors for aflatoxin B1 (AFB1) detection in food and beverage samples, without pretending to be exhaustive, we present an overview of the most important label-free and labeled sensing strategies. Simultaneous and competitive aptamer-based strategies are also discussed. The aptasensors are summarized in tabular format according to the detection mode. Sample treatments performed prior analysis are discussed. Emphasis was placed on the nanomaterials used in the aptasensors’ design for aptamer-tailored immobilization and/or signal amplification. The advantages and limitations of AFB1 electrochemical aptasensors for field detection are presented.

## 1. Introduction

Mycotoxins, a group of secondary metabolites produced by filamentous fungi, contaminate around 25% of the world’s agricultural products during different stages of their economic life: growth, harvest, storage, and processing [1]. They represent a large group of mycotoxins, but the most predominant are nivalenol, 3-acetyl-deoxynivalenol, 15-actyl-deoxinivalenol, ochratoxin (OTA), aflatoxin B1 (AFB1), and fumonisin B1 (FB1) [2,3]. AFB1 is probably the most widely known and definitely the most toxic of its group. It is known to have the ability to bind cell DNA and increase the risk of liver cancer, even if it might be found just in traces. Considering this, together with its mutagenic, teratogenic, immunosuppressive, and carcinogenic mechanism of action, AFB1 has been included by the International Agency for Research on Cancer in the number one group of carcinogens [2,3,4]. Without a doubt, there is a need to keep AFB1 concentrations under control and the contamination at a minimum level. Therefore, many local food and health agencies set a limit for AFB1 in different food products. Usually, the limits are between 0.05 and 20 ng/mL for cereals and cereal-derived products, where the risk of contamination is the highest [5]. But in some countries, like the USA, the limits can be set by the Food and Drug Administration (FDA) even for corn or peanuts [6].

In order to keep control of AFB1 levels, a continuous and rigorous analysis of its concentration in food products is needed. Traditional methods like high-performance liquid chromatography, mass spectrometry, or a combination of both [7,8,9,10] are reliable with very good sensitivity and selectivity, but they are very expensive, time-consuming, and lack the ability to analyze a high number of samples in different environments [11].

A good alternative to traditional methods could be electrochemical sensors. An electrochemical sensor stands for the sensor in which the transducer is an electrode surface. Some essential advantages derive from this, like (i) the use of the electron for signal acquisition, which is considered a green model for analytical applications, with minimal or no waste generation, (ii) the ability of miniaturization in order to develop portable devices, (iii) the short response time, and (iv) the low production costs [12]. The use of a recognition element in the development of an electrochemical sensor increases its selectivity. When the recognition element is represented by a biological-derived compound like an aptamer, antibody, bacteria, enzyme, or whole cell, it is called a biosensor. The use of a bioelement as a recognition system leads to high selectivity for the target analyte mainly because of the specific interaction between the analyte and the receptor. More importantly, the specific interaction prevents signal interference from other matrix components, which could modify the sensor’s response to a more specific one [13].

Aptamers are short single-stranded RNA or DNA sequences, selected by the SELEX (Systematic Evolution of Ligands by Exponential enrichment) technique from synthetic oligonucleotide libraries containing up to 10^15^ different sequences. Since the first SELEX selection described in 1990 [14,15], a significant number of aptamer sequences with high affinity and selectivity toward small molecules have been reported. The current SELEX technologies rely on the core process of the iterative in vitro selection of sequences based on successive key steps such as the binding, partitioning, and amplification of the bound sequences, but new features were introduced to reduce the selection time and costs and improve the aptamers’ stability and efficiency [16,17].

Compared with other biological recognition elements, they come with some advantages like good stability, wide target range, easy synthesis, low development cost, and most importantly, a good capacity for structural modification according to the scientific needs. As already stated, their ability to form different structures like hairpins, pseudoknots, convex rings, and G-quadruplexes leads to an increased conformational recognition of the targets similar to an antigen–antibody reaction [18]. In electrochemical sensing, their role is to bind different specific targeted molecules, from large to small organic molecules, such as proteins, biomarkers, xenobiotics, and toxins, to name a few [19]. When used in biosensors as bio-recognition elements, aptamers can be used directly linked to the transducer, both as a single-target or multi-target probe (labeled or label-free). Another possible approach is given by the possibility to use aptamer–target interaction to indirectly activate on–off devices in which the interaction with the analyte or the aptamer itself inhibits certain reactivity. Moreover, aptamers are subjected to significant conformational change caused by the interaction with the analyte that can be used as a recognition parameter when combined with appropriate transducers [20].

Ever since the development of the first AFB1-specific aptamer in 2012 (Patent > PCT/CA2010/001292), more and more AFB1 electrochemical aptasensors have been published, showing at the same time the need and the continuous interest toward fast and reliable AFB1 detection.

**Figure 1:**
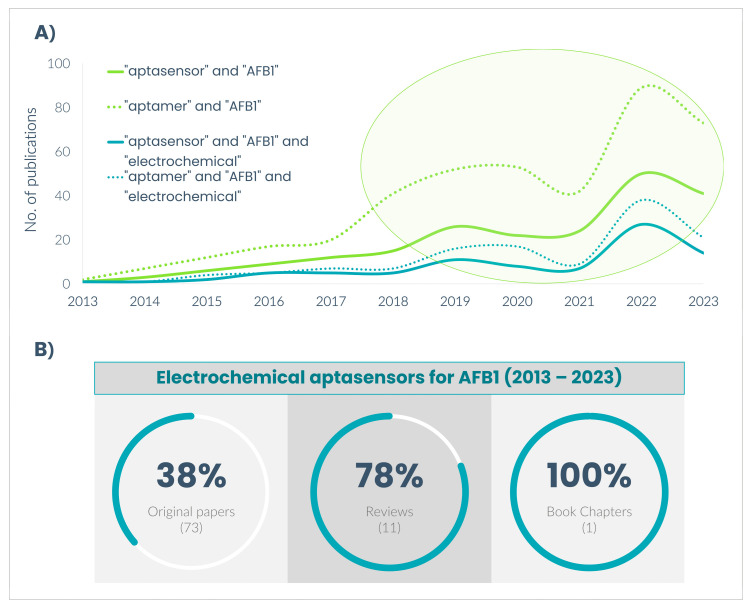
(**A**) Literature report of AFB1 detection based on aptamers (2013–2023) and (**B**) the distribution of electrochemical methods out of all reported methods (Scopus database).

According to the Scopus database, 400 papers (original papers and reviews) were found using the terms “aptamer” and “AFB1” and 206 papers when using the terms “aptasensor” and “AFB1”. The publication trend over time saw a considerable decrease in 2021 due to the impact of the COVID-19 pandemic, but rapidly recovered by the end of 2022, outgrowing any values reached by then (Figure 1A). Among these, the electrochemical aptasensing of AFB1 accounts for 38% of original papers and for 78% of review papers (Figure 1B). In addition, regardless of the combination of keywords used in the literature search, the number of publications in 2022 almost doubled compared to the pre-pandemic years. Reviews reporting the detection of toxins [21,22], mycotoxins [18,23,24], or aflatoxins [25,26,27,28] based on analytical methods in general [21,27,29], or specifically by optical [22,23,26,28] and/or electrochemical [18,22,23,25] transduction methods, have been published relatively recently (2022–2023). However, only a few of these have devoted the discussion over the use of aptamers for aflatoxin detection [21,24,26], but none specifically on AFB1 via electrochemical detection strategies. For this reason, an overview of the latest progress in AFB1 electrochemical detection based on aptamers (2018–2023) is of high interest. This review will also provide insights into the challenges and prospects of electrochemical aptasensors for the *on-site* detection of AFB1.

**Figure 2:**
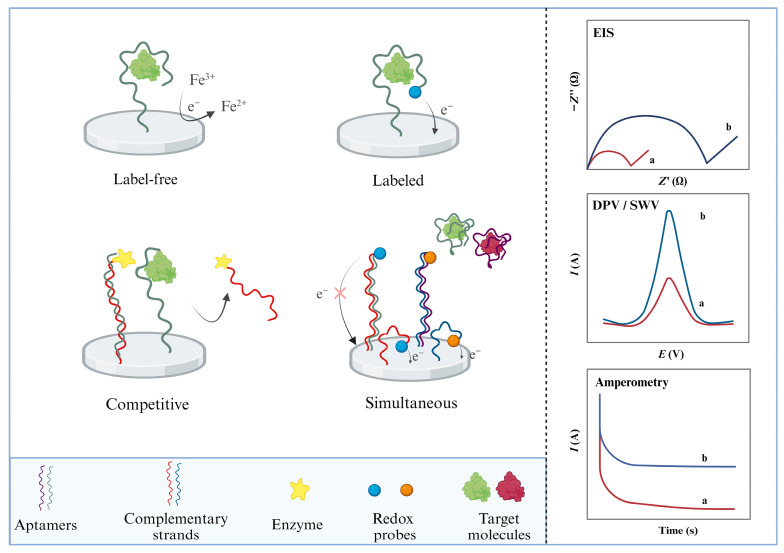
Schematic representation of main electrochemical aptasensor designs (label-free, labeled, competitive, and simultaneous) and the detection methods (EIS—electrochemical impedance spectroscopy; DPV—differential pulse voltammetry; SWV—square wave voltammetry; amperometry, where “a” and “b” represent the signal change upon target interaction). Created with Biorender.com.

The following sections present the most relevant approaches reporting AFB1 aptamer-based electrochemical detection, classified according to the detection mode: label-free and labeled assays (Figure 2), out of which competitive and simultaneous assays were treated as separate sections due to their possible future perspectives. Exceptional examples of aptamer DNA nanoarchitectures are presented. This review also examines the possible applications, challenges, and prospects of electrochemical aptasensors for the *on-site* detection of AFB1.

## 2. Food Contamination with AFB1 (Incidence, Toxicity, and Legal Limits)

Aflatoxins, among the numerous mycotoxins discovered so far, have emerged as a subject of profound scientific interest. This is due to their remarkable toxicity and carcinogenic potential, with some aflatoxin subtypes being officially included in Group 1 carcinogens by the International Agency for Research on Cancer [30]. These molecules are metabolites of the fungal species *Aspergillus flavus* and *Aspergillus parasiticus*, which are often found in food and feed, primarily in peanuts, corn, and rice. The tropical and subtropical climate favors the development of both fungi and toxins, as it provides the optimal temperature and humidity. However, their presence in food is not solely determined by geographical location but may also arise in response to inadequate conditions of storage, transportation, or crop processing [31,32].

When consumed in high quantities, aflatoxins can precipitate acute toxic responses, leading to a condition known as aflatoxicosis. Aflatoxicosis carries the potential for fatal consequences, primarily through the induction of hepatic failure. Additionally, aflatoxins can also have chronic toxic effects, manifesting as carcinogenicity affecting various organs, with a predilection for the liver and kidneys [33].

Under the action of cytochrome P450 enzymes, especially CYP3A4, the epoxidation of AFB1 occurs, resulting in the formation of 8,9-epoxy-AFB1. Only the 8,9-*exo*-epoxy-AFB1 isomer exhibits mutagenic activity by forming adducts with DNA or proteins, while the *endo* isomer is non-toxic. Due to interindividual variability in the expression of cytochrome P450 enzymes, some individuals may be more susceptible to the formation of the toxic metabolite, thus having an increased risk of developing liver carcinoma [34].

Hence, the presence of AFB1 in food items has been acknowledged as a serious food safety concern, resulting in the implementation of certain maximum levels by authorities. Permissible thresholds for AFB1 levels vary across different regions and countries around the world, with the maximum permissible for most consumer items in the European Union being 2 ng/mL, 10 ng/mL in Japan and Korea, and 20 ng/mL in the United States [35,36,37].

## 3. Nanomaterials Used for Electrode Modification

One of the biggest challenges in biosensors’ development is to obtain a very selective method and a good signal amplification to enable a low limit of detection (LOD). Since traditional electrochemical cells lack selectivity and sensitivity, different nanomaterials must be grafted on the surface of the working electrodes [38]. Hence, due to their functional groups and ease of conjugation, one key role relies on anchoring the aptamer sequences at the electrode surface. Depending on the application envisaged, the size and functionalities may vary.

Nanomaterials are divided according to their size and structure into zero dimensional (0D) nanomaterials like nanoparticles [39], QDs [24] or nanoclusters [40], 1D nanomaterials, such as nanotubes [41], nanowires [42], and nanorods [43], and 2D nanomaterials where the most representative examples are solid crystalline 1D nanomaterials that can form strong interlayer interactions [44]. In addition, they can form interactions with other layers of different nanomaterials, obtaining better physicochemical properties and new optical and electrochemical features, extremely helpful in DNA sensing [22,45]. The last and most recent category, 3D nanomaterials, is a combination of 0D–2D materials. Most often, they are developed by combining different conventional nanomaterials with a nanocomposite, or by the self-assembly of different nanoparticle systems [46].

Analyzing their composition, different types of nanomaterials have been used in the last couple of years for toxin detection and specifically for AFB1 electrochemical detection. Conductive polymers are widely known for their good electric conductivity acquired through redox moieties. Moreover, controlled polymerization allows for the formation of polymer structures that are not only similar on the entire WE surface, but also make possible the incorporation of different nanomaterials, which improves the accuracy [47]. Polyheteroaromatic polymers, such as polypyrrole, polyaniline (PANI), and their derivatives, have attracted a lot of attention lately because of their possible application toward electrochemical detection [48,49,50]. Being one of the most versatile conductive polymers, PANI was used in order to increase the electroconductive properties of a platform developed for AFB1 aptasensing in wine with very low LODs, down to 0.002 fg/mL [51].

Metallic nanoparticles (MNPs) are nanosized metals with sizes ranging from 10 nm to 100 nm. The development of novel MNPs with different structures, shapes, and dimensions has increasingly attracted the attention of the scientific world in the last few years. They have unique properties, such as high electrical conductivity, large surface-to-volume ratio, increased biocompatibility, and the ability to increase the catalytic activity of the electrochemical platform. All of these provide a huge space for MNPs in improving the sensors’ overall performance [52].

Carbon-based nanomaterials have some advantages compared with metal-based nanomaterials, including a lower price and higher surface area, thus leading to the possibility of the immobilization of a high amount of biological compounds. They are also very versatile, and a high number of structures can be obtained with specific characteristics [53]. From the carbon-based category, the most used are graphene oxides, which maintain their oxidized (GO) form, and reduced graphene oxides (rGOs), which have slightly modified electrochemical properties. GOs distinguish themselves by having distinctive properties like a greater hydrophilicity due to the high number of reactive oxygen groups, easily controllable electronic properties, wider potential window, and negligible residual current [54]. Beheshti-Marnani et al. developed an easy-to-use, electrochemical aptasensor for AFB1 detection, using only rGO nanosheets immobilized on the surface of a glassy carbon electrode (GCE) and an amino-labeled aptamer [55].

Nanomaterials play multiple roles in aptasensors’ design, but sometimes the sensitivity obtained by using different combinations of nanomaterials fails to meet the detection of AFB1 in trace amounts. Aptasensors based on multiple amplification strategies could be the solution to achieving the sensitivity goals. These include the use of enzymes, nanozymes, and DNAzymes [56]. An alternative could be the utilization of an exonuclease-assisted signal amplification strategy, usually promoted by exonuclease I (EXO I) [57,58], EXO II, or EXO III [59,60]. Zheng et al. performed a two-round signal amplification using EXO III and telomerase to obtain an LOD as low as 60 ag/mL using a voltametric technique and an AFB1 aptasensor. On the first amplification round, the telomerase was amplified to elongate the ssDNA probes on the gold nanoparticles deposed on the working electrode surface, leading to a “signal-off” electrochemical response. On the second step, the EXO III amplification had the role of hydrolyzing the 3′-end of the dsDNA right after the interaction with the AFB1 target, leading to a release of bound AFB1 back into the solution, starting a new recognition–amplification cycle [60]. The review of Rhouati et al. presents an in-depth analysis about enzyme-assisted amplification strategies in electrochemical aptasensors [56].

## 4. Aptamers for AFB1

Table 1 outlines the main aptamers for AFB1 used in the design of the electrochemical aptasensors evaluated in this review (48 studies), detailing their nucleotide content, length, affinity constants, original selection paper (or papers evaluating affinity constants of truncated versions), and the secondary structures. Notably, regardless of the detection strategy, the leading aptamer sequence is still the first patented aptamer for AFB1, denoted as Apt1 here, which was selected by Linda C. Le, Jorge A. Cruz-Aguado, and Gregory A. Penner from Neoventures Biotechnology LTD (London, ON, Canada) in 2012 [61]. Apt1 shares 60% of studies, followed by Apt 1 truncated versions 1 and 3, with 11% and 9%, respectively. However, to the best of our knowledge, only a few papers report the affinity constants of the aptamers and due to the considerable amount of the publications on AFB1 aptasensing, papers fail to report the source of the sequences used. This leads to misinformation and possible error generation, such as undesired sequence truncation or mismatches. For example, Apt 5 was selected by Cruz-Aguado and Penner for Ochratoxin A in 2008 [62]. No data were found concerning the affinity of the sequence towardsAFB1; instead, other papers might have reported it. The secondary structures were predicted by the Mfold webserver [63] by setting up the concentrations of 0.1 M Na^+^ and 0.05M Mg^2+^ and the temperature to 25 °C. Most aptamers present a central stem–loop secondary structure with a number of base pairs ranging from 1 up to 7. Truncated versions of the primary Apt1 retained good affinities toward AFB1, suggesting that the stem–loop has a great contribution in the interaction with the target. As not all sequences were found in the literature to compare the dissociation constants, Gibbs free energy (Δ*G*) could be an indicator of the nature of the aptamer–AFB1 interaction and the affinity of the aptamer. Generally, the lower the ΔG value, the higher the affinity [64]. Still, among all, Apt1 and its truncated version 2 have exhibited the lowest values.

## 5. Food and Beverage Samples Treatment

Because of the good thermal and chemical stability of AFB1, different pretreatment steps have been reported, mostly among the traditional sample pretreatment methods, such as liquid–liquid extraction, centrifugation, and filtration. Generally, the samples are treated with organic mixtures to ensure the extraction of AFB1, such as methanol:water (*v*/*v*) (6:4) [51], (7:3) [42,69], (8:2) [70] or acetonitrile buffer (*v*/*v*) (7:3) [71]. A mixing step of about 30 min [69] up to 5 h and a further centrifugation and filtration through 0.22 μm or 0.45 μm filter membranes are usually performed. However, limitations such as a high consumption of organic solvents, time-consuming steps, poor selectivity, and low recovery and enrichment factors are encountered. Solid-phase microextraction, magnetic solid-phase extraction, dispersive solid-phase extraction, and matrix solid-phase dispersion extraction can overcome the disadvantages of traditional methods. Materials with efficient adsorption properties, high and easily tailored surface areas, such as metallic organic frameworks (MOFs), covalent organic frameworks (COFs), molecularly imprinted polymers (MIPs), carbon-based nanomaterials, and aerogels, become of interest in the pretreatment step of foodstuffs [72]. MOFs, as emerging advanced materials, may serve both as a pretreatment tool and electrode surface modifier [73]. To improve the enrichment efficiency, magnetic particles can be incorporated into the MOF structure [74]. Enhanced selectivity can be achieved using biological (antibody) or biomimetic molecules (aptamer or molecularly imprinted polymers). Purification steps with immunoaffinity columns containing antibodies specific to AFB1 ensure the pre-concentration of and reduction in the matrix effect [75]. Although aptamers are stable in different media, aqueous mixtures are of election for electrochemical measurements, especially at screen-printed electrodes. Phosphate or TRIS buffers are usually used for the dilution of extracts prior to measurements. Corn flour and oil [42], wheat flour, peanuts [51,75,76] and peanut oil [42], beer [71], and wine [71,77] are commonly tested for the presence of AFB1. The standard addition method is frequently reported with spiking AFB1 solutions in the pg-ng/mL concentration range.

Advanced and efficient sample pretreatment methods for electrochemical sensing are still a major requirement nowadays. Recent reviews [72,78] report advanced methods for food sample preparation, but, generally, the analyte determination is further performed via conventional chromatographic methods. Sufficient enrichment of and efficient reduction in the matrix interference must be achieved to improve the accuracy and sensitivity of the electrochemical assays to address the food analysis requirements.

## 6. Applications of Electrochemical Aptasensors for AFB1 Detection

The increased need for fast, accurate, and feasible detection methods of mycotoxins turned the scientific world’s attention toward biosensing. Novel detection methods had to be found and applied in real samples, with very low LODs and high selectivity.

Aptamers have been incorporated into detection devices, such as biosensors, with the role of a biological recognition element and used more and more often for the detection of mycotoxins and, more specifically, AFB1. When the electrode is the transducer of the aptasensor, the detection tool emerges as the foundation stone toward portability. The electrochemical signal is generated directly or indirectly upon the aptamer–target interaction and amplified by different strategies. The past few years have witnessed a particular growth in the development of novel aptasensing approaches, electrochemical materials, and methods of aptamer incorporation into the sensing design. This review presents the latest publications regarding AFB1 electrochemical aptasensing, highlighting the most efficient aptamer sequences and analytical figures of merit of the aptasensors, as summarized in Table 2. The main electrochemical designs are schematically presented in Figure 2, whilst the most particular designs are discussed and illustrated. Challenges and possible solutions are discussed.

### 6.1. Label-Free Assays

Label-free approaches enable an electrochemical signal upon the changes that occur after the aptamer–target binding event that can be monitored at the electrochemical interface with the aid of an external redox probe or the intrinsic redox activity of the aptamer–target complex [116]. As a result, the difficulty in obtaining an aptasensor with a very good sensitivity and selectivity is increased. In these cases, the electrochemical platform has a very important role in order to obtain a good signal amplification and to allow a high ratio of aptamer immobilization [117]. Nevertheless, label-free assays also have some important advantages compared with the labeled ones. They allow for real-time quantification thanks to the capacity of kinetic monitorization of the receptor–target interaction [116] and the lack of labeling leads to lower development costs [118]. Most importantly, the binding affinity of the sequences would be unaffected by an extra labeling process [119] as the aptamer–target interaction is mostly monitored by non-invasive electrochemical techniques, such as EIS [120].

Multiple label-free electrochemical aptasensors based on carbon nanotubes [82,87], graphene oxide [80], reduced graphene oxide [55,77,82,85,87], AuNPs [42,76,77,87] or Au-based materials [51,83,86,90], polymers [49,77,80], and MOFs [79] have been reported recently.

**Figure 3:**
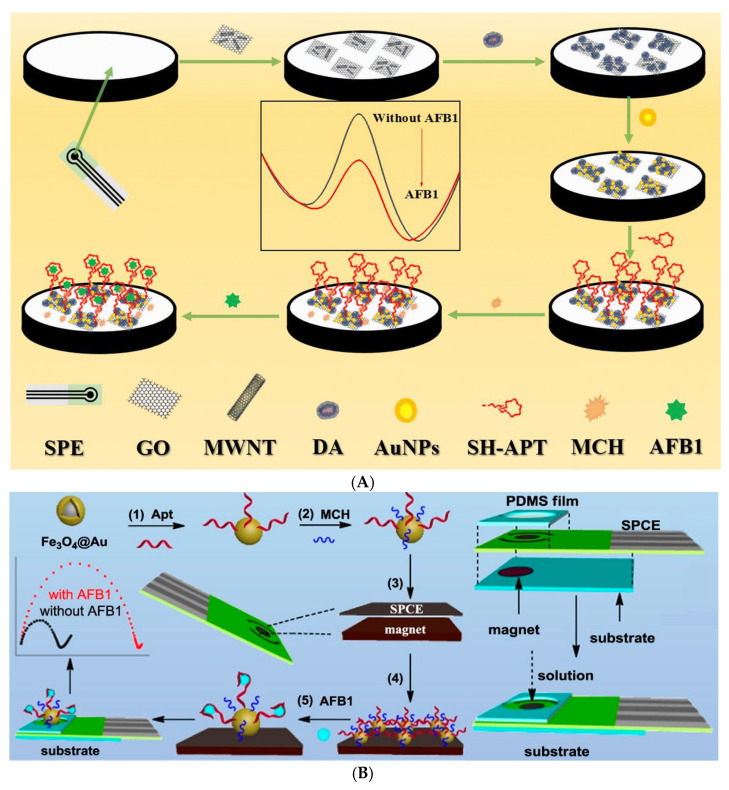
Label-free electrochemical aptasensor based on (**A**) COOH–GO–COOH–MWNT/pDA/AuNPs (reprinted from [87] with permission from the Royal Society of Chemistry) and (**B**) Fe_3_O_4_@Au-Apt nanospheres (reprinted with permission from [51]).

As described above, AFB1 has a very harmful effect even in extremely low concentrations; therefore, it is almost mandatory to be able to detect it even in traces. To achieve this need, Wang P. et al. developed a very sensitive label-free electrochemical aptasensor based on Apt1 truncated v3, with the lowest LOD from this category to the best of our knowledge. Starting from an SPCE and using a combination of different nanomaterials like GO, MWCNT, and AuNPs, the authors report the detection of AFB1 as low as 15.4 ag/mL, with a wide dynamic range of 0.1–10^5^ fg/mL, allowing for the detection of the toxin in traces but also in higher concentrations (Figure 3A) [87]. The downside of such an elaborate aptasensor is that the immobilization of a high number of nanomaterials can be time-consuming and that the 1 h interaction between the aptasensor and the analyte increases the risk of unspecific adsorption in complex matrixes, like the milk products used to demonstrate its application in real samples. Hence, as the maximum levels of AFB1 established by the competent legislation are in the ng/mL range, there is no need to lower the detection limits by 9 orders of magnitude.

In a different approach that could help save time, Apt1 was firstly immobilized on Fe_3_O_4_@Au nanospheres using the Au-SH affinity reaction and the remaining free Au sites were blocked using the -SH group of 6-Mercaptohexanol (MCH) in order to avoid unspecific adsorption in complex matrixes. In the end, the newly developed Fe_3_O_4_@Au-Apt nanosphere was dropped and kept on the surface of an SPCE using the magnetic field. After the interaction with the AFB1 solution, the analyte was detected in concentrations as low as 15 pg/mL (Figure 3B). Since the number of products that can be contaminated with AFB1 is quite high, with different possible concentrations and most importantly different matrix and chemical environments, in AFB1 electrochemical detection, one solution cannot fit all. Therefore, aptasensors have been developed specifically for the detection in some food samples: alcoholic beverages like wine or beer [77], peanut and corn oil [42], and even soy sauce [89].

Since, in some cases, there is also a need to detect the toxin not before but after its ingestion, it is important to be able to detect the toxin in biological samples fast, accurately, and in low doses. Even though there are still many steps to be made in order to achieve all three characteristics, there have already been label-free biosensors published detecting AFB1 in biological samples. One example belongs to M. Roushani et al. who developed a simple Au nanorod on a GCE-based, label-free aptasensor. Importantly, the time of contact between the aptasensor and AFB1 solution was only 15 min; this leads to fast detection, a critical aspect for point-of-care testing, especially for identifying an acute toxic event. The aptasensor that has an LOD of just 0.09 pg/mL was able to detect AFB1 in human serum samples, showing promising results toward clinical applications [90].

### 6.2. Labeled Assays

The labeled-based aptasensing approaches consist of redox-active molecules that are tagged at one end of the aptamer (5′ or 3′). Labeled complementary sequences can be used in the design to undergo the analytical signal [121,122]. Labeled sandwich assays that contain multiple aptamers are not common for AFB1 detection as the target is relatively small.

A typical labeled electrochemical aptasensor contains a redox probe, such as ferrocene (Fc), methylene blue (MB), thionine (Thi), or anthraquinone (AQ), tethered at the aptamer sequences or complementary strands, respectively. Generally, the detection mechanism lies on the conformational changes that occur in the aptamer 3D structure upon aptamer–target interaction, which cause a change in the electrochemical signal of the redox probe by distance regulation or steric effect strategies [17,123].

The “signal-on” approach is encountered when the folding of the aptamer upon analyte addition determines the proximity of the redox probe to the electrode surface, consequently increasing the obtained electrochemical signal [101,110]. On the contrary, the “signal-off” approach entails a greater distance between the label and the electrode after the aptamer–target interaction, thus decreasing the electrochemical signal [111].

**Figure 4:**
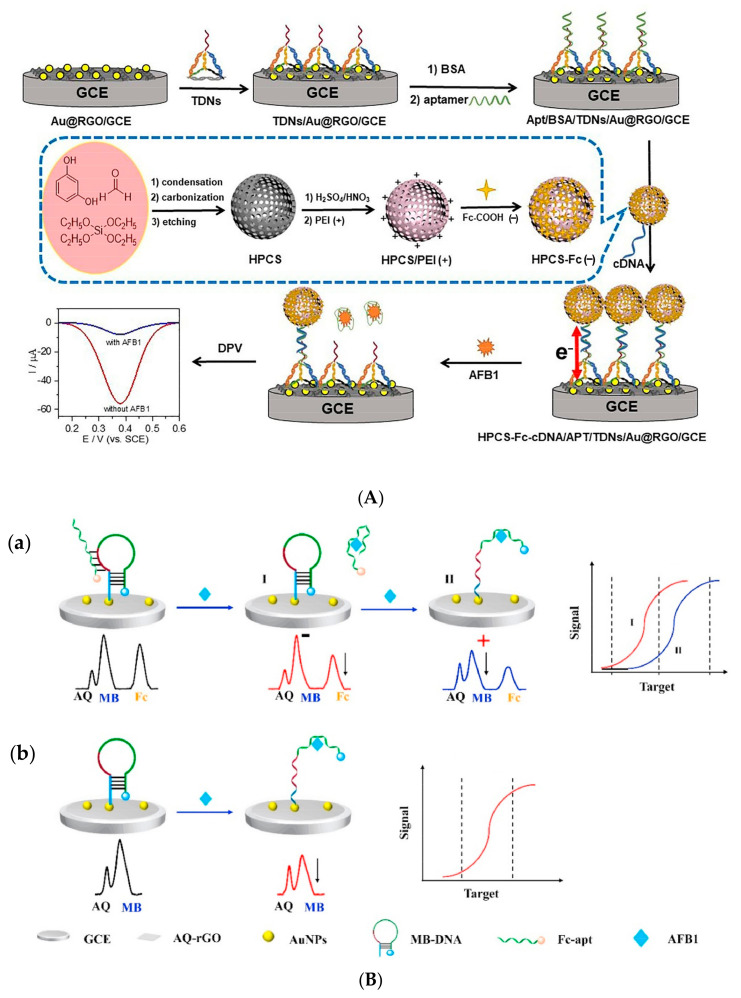
(**A**) Labeled aptasensor based on Fc signal tag incorporated in HPCS through a layer-by-layer assembly and tetrahedral DNA nanostructures (TDNs). Reprinted with permission from [96]. (**B**) Ratiometric-labeled aptasensor following (**a**) the signal reaction process of HP and linear-HP (I and II) on a single sensing interface of the aptasensor and (**b**) the reaction process of HP on the surface of the aptasensor. Reprinted with permission from [98].

Fc is widely used as a signal reporter in electrochemical aptasensors due to its intrinsic redox activity. Although far from being the optimal label due to its low solubility and poor adsorption, nanomaterials with increased electronic transport properties and large surface area can improve the features of Fc in electrochemical aptasensing [124]. Multiple Fc-labeled electrochemical aptasensors based on carbon nanotubes [92], reduced graphene oxide [93,94,96,97], AuNPs [94,95,96,100], and MOFs [103] have been reported recently. A recent study reported the use of hollow porous carbon spheres (HPCSs) tagged with a Fc label and tetrahedral DNA nanostructures for the sensitive detection of AFB1 in the range from 1.0 × 10^−2^ pg/mL to 100.0 μg/mL with an LOD of 0.033 pg/mL (Figure 4A). Although the LOD is considerably low, the preparation of the aptasensor is quite laborious and requires multiple hours for platform preparation (>38 h), aptasensor development (7.5 h), and the assay itself (50 min) [96]. Most of the studies report the use of Apt1 [92,93,94,95,96,97,98] with LODs in the pg/mL to ng/mL range, whereas Apt3 version 1 [103] and Apt1 v1 reversed [100] seem to enable more sensitive aptasensors with LODs of 4.81 fg/mL and 12.00 fg/mL, respectively.

MB is one of the commonly used redox reporters and it is frequently labeled at the 3′-end of the aptamer strand [67]. However, an interesting approach uses the intercalation of the MB label at a specific thymine internal site of 26-mer Apt1v4, derived from the Apt1 sequence. The aptasensor based on a “signal-on” distance regulation strategy allowed for the detection of AFB1 by square wave voltammetry (SWV) [101]. By contrast, when two nucleotides were cut at both ends, resulting in the 22-mer Apt1 truncated v5, a LOD of 1.87 pg/mL (6 pM) was obtained [67]. Nevertheless, to prove the selectivity, all aptasensors were subjected to interferents from the class of mycotoxins.

Currently, the use of ratiometric biosensors has received attention toward mycotoxin detection [18]. Ratiometric-labeled electrochemical aptasensors use at least two redox reporters simultaneously to reduce the matrix effect in real samples and increase the sensitivity, reliability, and reproducibility of the assays by built-in signal corrections. The ratio of the electrochemical signals generates the output signal that is further correlated to the analyte concentration, either by a signal on–off mode when both receptors are labeled at the aptamer strand [98] or by a single signal/reference mode when at least one receptor is incorporated in the aptamer immobilization nanomaterial and acts as an internal redox reference [93,94,97,98]. A remarkable ratiometric aptasensor using both Fc and MB aptamer labels and AQ as the internal reference embedded in rGO enabled the detection of AFB1 as low as 0.01 pg/mL (Figure 4B) [98]. Nevertheless, the affinity of the aptamers suffers changes upon modification with redox tags as anchoring sites may become limited or unavailable to interact with the target molecules [125].

### 6.3. Competitive Assays

It is worth highlighting that competitive and simultaneous approaches do not stand alone as detection modes and are often based on labeled strategies. However, competitive assays involve the use of a cDNA strand to compete with the target molecules toward the interaction with the aptamer. The aptamer–target complex must have a higher stability than the dsDNA formed by hybridization with the cDNA to have an efficient detection system. The detection mechanism is mostly based on a target binding-induced aptamer or complementary strand displacement [121]. The signal amplification strategies in competitive aptasensors lie more on enzymatic assays (DNAzyme [69], alkaline phosphatase [50], Exo I [57]) or hybridization chain reaction (HCR) [108,111], but nanomaterials such as AuNPs [104,109,112,113], polymers [50], and MOFs [104,105] have also been reported.

**Figure 5:**
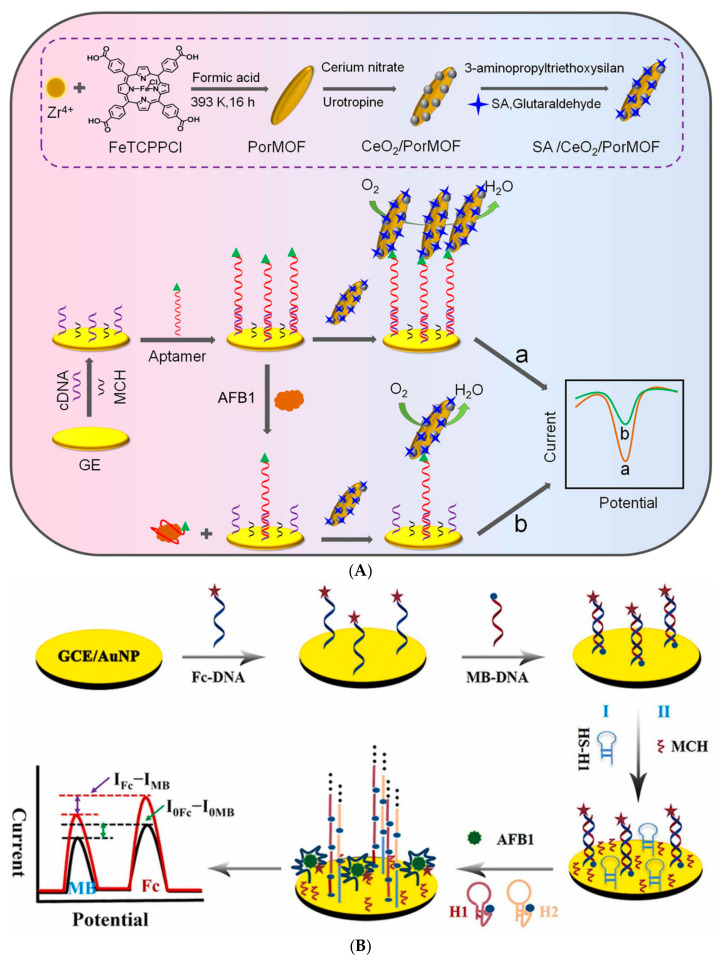
(**A**) CeO_2_/Fe-porphyrinic MOF composite aptasensor based on aptamer–target displacement mechanism. Reprinted with permission from [105]. (**B**) Ratiometric DECA method based on HCR signal amplification. Reprinted with permission from [109].

Zhang et al. developed a label-free competitive aptasensor using an interesting signal amplifier probe. Cerium oxide nanoparticles were anchored on a porphyrinic MOF, subsequently modified with streptavidin (SA/CeO_2_NPs/PorMOFs). The cDNA was immobilized onto GE through Au-S bonds, while the unreacted gold surface was blocked with MCH. Furthermore, the 3′-biotinylated Apt1 was incubated and bound to the complementary strand. As depicted in Figure 5A, the absence of AFB1 allowed biotin and streptavidin to interact, therefore bringing the CeO_2_/PorMOFs near the electrode surface and determining a substantial oxygen reduction current. In contrast, when the target was present, the aptamer exhibited a preference for binding to AFB1 and, consequently, the lack of the signal probe resulted in a decrease in the analytical signal. The CeO_2_NPs increased the electrochemical current by being a catalyst for oxygen reduction, thus lowering the LOD of the aptasensor down to 9.37 fg/mL. With a sample incubation time of one hour, this aptasensor was successfully tested in complex matrixes, such as peanuts and milk, showing great reproducibility and accuracy [105].

In the competitive assays selected for this review, the most employed aptamer sequences were Apt1 and Apt1 truncated v3. Interestingly, only a few studies have reported the use of disposable transducers, namely SPCE [50] and SPGE [106], to facilitate the analysis for *on-site* applications. A label-free semiconductor QDs-based ratiometric electrochemical aptasensor was developed in several steps including endonuclease cleavage, magnetic separation, and dissolution in acidic media. Finally, the electrocatalytic oxidation of Pb^2+^ and Cd^2+^ by SWV stripping measurements was correlated inversely with the AFB1 concentration, as low as 4.5 pg/mL [107].

In the work of Liu et al., the novelty consists of a double “signal on” approach, both from a Fc-tagged aptamer and MB-tagged cDNA and DNA nanowire to work as a labeled competitive ratiometric sensor (Figure 5B). GCE was first modified with AuNPs, serving as a specific binding surface for Fc-Apt and MB-cDNA, which formed a dsDNA. Subsequently, an HP functioning as a substrate for the HCR was introduced onto the surface and MCH was immobilized to block any possible uncovered active sites. In parallel, the AFB1 samples were prepared with a mixture of an MB-labeled auxiliary HP1 and HP2. In the absence of AFB1, the sensor retained its original electrochemical signals from both Fc and MB. When AFB1 was present, it bound to Fc-Apt, leading to the release of MB-cDNA and triggering the unfolding of HP, which initiated an HCR between auxiliary HP1 and HP2. This reaction involved the binding of the two hairpin strands to form a long MB-tagged DNA nanowire. As the nanowire was formed, it generated an increased electrochemical signal in correlation with the quantity of MB. Simultaneously, the conformational change in Fc-DNA brought it closer to the electrode surface, facilitating electron transfer, which enhanced the electrochemical signal of Fc. Using HCR as a signal amplification strategy was highly effective in this case, providing a low LOD of 37 fg/mL. Moreover, the aptasensor’s functionality was proven by rapidly detecting AFB1 in moldy samples of peanut and cereals within just 20 min, setting a promising precedent for future *on-site* AFB1 detection [109]. Another labeled assay reports the detection of AFB1 upon 5 min incubation of the sample and possible regeneration and reuse of the aptasensor [111].

A similar competitive format using a label-free HCR amplification method has enabled the detection of AFB1 as low as 2.84 fg/mL [108]. Nevertheless, the signal amplification tools considerably add value to the aptasensor’s sensitivity, but overall increase the level of complexity in the work of principle of the sensor.

### 6.4. Simultaneous Aptasensing of Toxins

**Figure 6:**
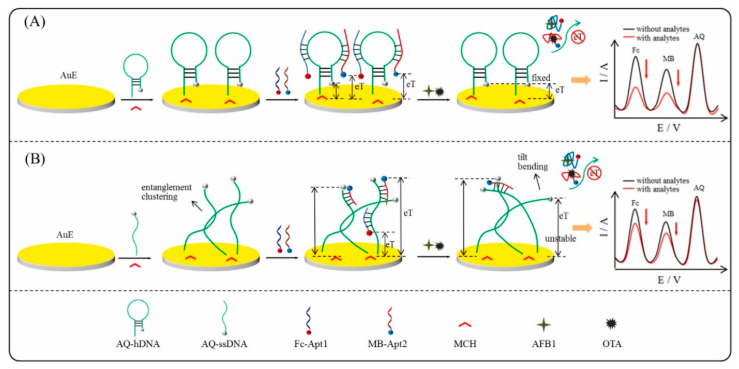
Simultaneous electrochemical analysis of AFB1 and OTA by a competitive ratiometric approach using Fc and MB labels as target indicators and AQ as a reference signal. The schematic representation of the hDNA (**A**) and ssDNA (**B**) configurations of the capture probe. Reprinted with permission from [115].

As contaminated foods may contain multiple toxins, selective and sensitive multi-target analysis becomes mandatory to fit the requirements nowadays of biotechnological advances. Dual electrochemical aptasensors for simultaneous AFB1 and OTA detection have been reported [114,115]. Both examples have reported the use of the Apt1 sequence and enabled LODs in the pg/mL level. Zhu et al. have developed a dual-ratiometric aptasensor using a Fc-labeled Apt1 and MB-labeled OTA aptamer. The aptamer strands specific to each target were immobilized by hybridization with a cDNA capture probe, owing to a hairpin configuration, grafted at the gold electrode surface. The cDNA was labeled with AQ and was used as a reference signal. The aptasensors enabled a “signal off” detection mode by a competitive approach, as the redox signals of Fc and MB decrease upon target addition. The ratio between Fc/AQ and MB/AQ was used to determine LODs as low as 4.3 pg/mL for AFB1 and 13.3 pg/mL for OTA. Interference studies were performed in the presence of other toxins, such as AFB2, OTB, FB1, and ZEN. The schematic representation of the aptasensor’s design and importance of the hairpin configuration of the capture probe are depicted in Figure 6.

## 7. Conclusions and Future Perspectives

This review aimed to present a short overview of the latest advances in electrochemical aptasensing strategies toward AFB1. The literature was selected mainly focusing on label-free and labeled aptasensors, but also competitive and simultaneous assays published in the time frame of 2018–2023. Particular strategies and novel materials were also highlighted. In addition, the aptamer sequences used in the sensor designs and the original selection sources were summarized in Table 1. In recent years, papers have reported the use of several aptamer sequences, but failed to report the affinity constants or original sources. More attention must be devoted when selecting the aptamer strands from other aptasensor papers due to possible error generation.

Although electrochemical approaches enable fast, highly sensitive, and selective detection tools, efforts have been made to lower the LODs of the developed electrochemical aptasensors to meet the values under the maximum accepted levels for AFB1.

Interestingly, although aimed at simple, fast, and *on-site* analysis, only a few electrochemical aptasensors analyzed in this review could pass the portability and/or ease-of-preparation tests and/or facile sample pretreatment steps. Therefore, many more optimizations must be performed in this direction as well.

There is still a large gap from the basic research to implementation in real scenarios. To the best of our knowledge, there is no commercially available electrochemical aptasensor for AFB1 or any other type of sensor to overcome the prototype phase. To this end, some crucial issues must be solved prior to use in regulated routine analysis as complementary to laboratory-based methods. In addition to long-term stability, and simple and efficient food extraction protocols, simultaneous screening becomes strongly required for *on-site* applications. Regardless of the features of electrochemical aptasensors, limitations related to substrate effects, low stability, non-specific adsorption, and electrolyte and temperature dependency are to be mentioned.

Interestingly, although aimed at simple, fast, and *on-site* analysis, only a few electrochemical aptasensors analyzed in this review could pass the portability and/or ease-of-preparation tests and/or facile sample treatment steps. Therefore, many more optimizations must be performed in this direction as well.

Overall, electrochemical aptasensors hold great value for the implementation of decentralized screening tools, to aid in the lower volume of samples analyzed by laboratory-based methods and reduce costs. Moreover, portability is required and easily employed by screen-printed transducers in combination with cost-effective and portable potentiostats.

The combination of aptamers with advanced materials to synergistically reinforce the electrochemical sensing properties is aimed at the identification and detection of traces of AFB1 in food and beverage samples. Efficient pretreatment steps and materials that allow for fast sample processing and detection are mandatory nowadays in the framework of food analysis and legislation requirements. Materials with efficient adsorption properties such as MOFs, COFs, MIPs, carbon-based nanomaterials, and aerogels can be used for removing the main impurities and selectively enriching AFB1 in the sample pretreatment steps. Hence, these materials can act both as pretreatment tools and electrode substrates to synergistically contribute to the aptasensors’ selectivity. For signal amplification, electrode modifiers such as carbon-based nanomaterials or Au nanostructures are mainly applied in the design of the aptasensors due to properties including an increased specific and electroactive area, providing a higher amount of immobilization sites for the aptamer strands and enhanced catalytic activity and electron transfer rates. Nevertheless, the fouling effect remains a critical issue to be addressed and multiple strategies based on polymers, hydrogels, peptides, or self-assembled monolayers can be adopted to prevent the unspecific adsorption of (bio)molecules.

Engineering efforts can contribute to the development of more robust and miniaturized sensing devices with multiplexed capabilities. Sensing platforms less prone to failure upon dramatic temperature, pH, or ionic strength alterations could represent possible solutions for apta-assays entering the market area. Biosensor arrays integrated into printed circuit boards dedicated to multiplexed detection in different pretreatment conditions could aid the improvement in the accuracy and reliability of electrochemical aptasensors. Hence, the simultaneous detection of different contaminants could ease the analysis process and food production flow.

Specific algorithms for data collection, processing, mining, and interpretation would be needed, whilst protocols could be useful for non-skilled personnel to follow the steps required for analysis completion. In addition to the above-mentioned critical steps, the commercialization of integrated electrochemical aptasensors will require intensive efforts in refining the analytical parameters and in consolidating electronics.

Coupled with smart electronics and advances in technology, aptasensors have a remarkable potential to pave a key milestone in biosensing technology.

## Figures and Tables

**Table 1:** Aptamer sequences used in the design of the electrochemical aptasensors selected for this review (48 studies from 2018–2023).

Name	AFB1 Aptamer Seq. (from 5′ to 3′)	Length	No. of Studies	*K*_d_ (nM) from Original Seq./Δ*G* (kcal/mol)	Secondary Structures Generated in Mfold Web Server [63]	Selection/Affinity Evaluation Ref.
Apt1	GTTGGGCACGTGTTGTCTCTCTGTGTCTCGTGCCCTTCGCTAGGCCCACA	50-mer	27	10 nM */−10.26	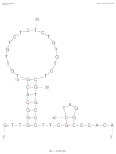	[61]
Apt1 truncated v1	GTTGGGCACGTGTTGTCTCTCTGTGTCTCGTGCCCTTCGCTAGGCCC	47-mer	5	Not mentioned/−10.26	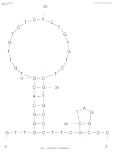	[61]
Apt1 truncated v1 reversed	CCCGGATCGCTTCCCGTGCTCTGTGTCTCTCTGTTGTGCACGGGTTG	47-mer	1	-/−9.96	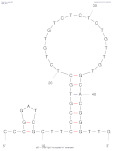	-
Apt1 truncated v2	CCCGTTGGGCACGTGTTGTCTCTCTGTGTCTCGTGCCCTTCGCTAGGGCCC	51-mer	2	Not mentioned/−11.35	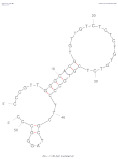	[61]
Apt1 truncated v3	GCACGTGTTGTCTCTCTGTGTCTCGTGC	28-mer	4	70 ± 2 nM * 30.9 ± 2.3 nM **^i^ 35 ± 4.2 nM **^ii^/−4.67	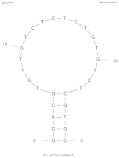	[65] * [66] **^i^ [67] **^ii^
Apt1 truncated v4	CACGTGTTGTCTCTCTGTGTCTCGTG	26-mer	1	49 ± 2 nM * 27.7 ± 2.4 nM **^i^ 94 ± 24 nM **^ii^ 21.8 nM ***/−2.16	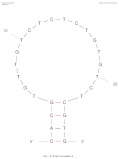	[65] * [66] **^i^/*** [67] **^ii^
Apt1 truncated v5	CGTGTTGTCTCTCTGTGTCTCG	22-mer	1	341 ± 20 nM ** ^i^ 498 ± 37.2 nM ** ^ii^/+1.23	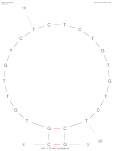	[66] **^i^ [67] **^ii^
Apt2	AGCAGCACAGAGGTCCAGTCGTATAAATTTACATGGCGTGCTACCGTGAA	50-mer	1	11.39 nM */−2.8	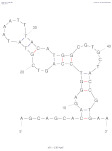	[68]
Apt3	TGGGGTTTTGGTGGCGGGTGGTGTACGGGCGAGGG	35-mer	2	-/−1.42	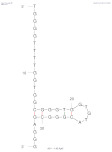	-
Apt3 truncated v1	TGGGGTTTTGGTGGCGGTGGTGTACGGGCGAGGG	34-mer	2	-/−1.43	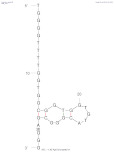	-
Apt3 truncated v2	TGGGGTTTGGTGGGTGGTGTACGGGCAGG	29-mer	1	-/+0.04	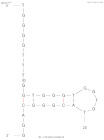	-
Apt4	GATCGGGTGTGGGTGGCGTAAAGGGAGCATCGGACA	36-mer	1	-/−0.61	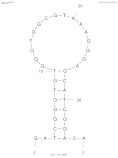	[62]

*K*_d_ determination technique: * fluorescence; ** isothermal titration calorimetry (^i^ first example, ^ii^ second example); *** surface plasmon resonance.

**Table 2:** Electrochemical aptasensors for detection of AFB1 (2018–2023).

Aptamer Name (Modifications Made in Direction 5′ à 3′)	Transducer Platform	Analysis Method	Dynamic Range	LOD	Interferents	Samples and Minimum Detectable AFB1 Concentration		Ref.
Label-free								
HO(CH_2_)_6_-S-S-(CH_2_)_6_-Apt1	GCE/rGO/MoS_2_/PANI@AuNPs/Apt/MCH	DPV	0.01–1.0 fg/mL	0.002 fg/mL	OTA, FB1	Wine	0.125 fg/mL	[77]
HS-Apt1	GCE/ZIF-8/AuNPs/Apt/MCH	EIS	10 pg/mL–0.1 µg/mL	1.820 pg/mL	AFB2, AFG1, AFG2	Corn oil, peanut oil	1 ng/mL	[42]
NH_2_-C_6_-Apt1	GCE/aminocaproic acid/Apt/AFB1/rGO	DPV	0.15–1.25 ng/mL	0.022 pg/mL	-	Human blood plasma and pasteurized cow milk	150 pg/mL	[55]
NH_2_-C_12_-Apt1	GCE/CuMOFs/GA/Apt/BSA	EIS	1.0 pg/mL–200.0 ng/mL	0.830 pg/mL	OTA, AFM1	Wheat flour	420 ng/mL	[79]
NH_2_-Apt1	GFE/PAA/Apt/GO	CA	1–20 ng/mL	0.130 ng/mL	OTA, AFG1, AFB2	-	-	[80]
Apt1-SH	SPCE/PANI Fe_3_O_4_@Au-Apt	EIS	20 pg/mL–50 ng/mL	0.015 ng/mL	FB1, AFB2, OTA	Peanut	0.5 ng/mL	[51]
NH_2_-Apt1	SPCE/PANI/GA/Apt	EIS	9.37–24.98 pg/mL	3.120 pg/mL	AFB2, OTA, OTB, ZEN	Pistachio nuts, cinnamon, cloves, corn, soybeans	18.720 pg/mL	[49]
SH-(CH_2_)_6_-Apt1	BDDE/AuNPs/Apt/MCH	EIS	31.22 pg/mL‒3.12 µg/mL	0.017 fg/mL	AFB2, AFG1, AFG2	Peanut powder	0.031 fg/mL	[76]
Apt1-NH_2_	ITOE/O-VMSF/Apt/BSA	DPV	3 pg/mL–3 µg/mL	0.002 ng/mL	ZEN, OTA, AFB2	Peanuts and corn	0.1 ng/mL	[81]
Apt1	ITOE/Au-hydrogel/MB-dsDNA	DPV	0.001 ng/mL–1000 ng/mL	0.800 pg/mL	-	Peanut, soil	5 ng/mL	[82]
SH-(CH_2_)_6_-Apt1	*µ*PAD/TDNs/Apt-Au@Ni-Co LDH NCs	DPV	0.2 pg/mL–100 ng/mL	0.071 pg/mL	OTA, ZEN, DON, T-2	Corn	0.1 ng/mL	[83]
Apt1 v1	GCE/hDNA-Apt/HP1+HP2	ACV	100 pg/mL–100 ng/mL	0.039 ng/mL	AFB2, ZEN, OTA, FB1	Corn, wheat, peanut, rice	1 ng/mL	[84]
NH_2_-Apt1 v1	GCE/Ti_3_C_2_Tx/GO-COOH-P4VP/Apt	DPV	0.01 ng/mL–50 ng/mL	0.003 ng/mL	AFG1, OTA	Grape juice, milk, soy milk	0.5 ng/mL	[85]
HS-(CH_2_)_6_-Apt1 v1	SPCE/AuNFs/APT/cDNA/CuNPs	DPV	0.031 fg/mL–31.22 pg/mL	2.107 ag/mL	AFB2, AFG1, AFG2	Peanut, rice, soy, millet, corn, chestnut, beer	0.312 pg/mL	[86]
SH-(CH_2_)_6_-Apt1 v3	SPCE/COOH–GO–COOH–MWCNTs/pDA/AuNPs/SH-Apt/MCH	DPV	0.1 fg/mL–100 pg/mL	15.140 ag/mL	AFB2, OTB, FB1, FB2, ZON, DON	Milk	0.1 pg/mL	[87]
SH-Apt2	GE/Apt/MCH	EIS	0.312–31.227 ng/mL	0.131 ng/mL	OTA, AFB2, AFG1, AFG2	Peanut	0.312 ng/mL	[88]
NH_2_-Apt3	GCE/PDDA-GNs/PS-COOH/BSA/Apt	EIS	0.001–0.1 ng/mL	0.002 ng/mL	OTA	Oil and soy sauce	0.1 ng/mL	[89]
NH_2_-Apt3 v1	GCE/AuNRs/Apt	DPV	0.31–78.07 pg/mL	0.090 pg/mL	AFM1, OTA, OTB	Human serum and rice samples	0.312 pg/mL	[90]
NH_2_–Apt3 v1	GCE/Cu_2_O NCs/Apt/MIP	EIS	50 fg/mL–40 pg/mL	0.012 pg/mL	AFG2, OTA, OTB, AFM1	Milk	2 pg/mL	[91]
Labeled								
NH_2_-(CH_2_)_6_-Apt1	SGPGE/NiCo_2_O_4_/MWCNTs/MXene/pDA/cDNA/MCH/Apt/TEMPO-COOH	DPV	2.5–200 ng/mL	1.890 ng/mL	AFB2, AFG1, AFG2, OTA, Vit B1, Citric acid, Glu, Gly, Na^+^, K^+^	Corn flour, corn residue	25 ng/mL	[92]
Apt1	GCE/THI-rGO/CS/Fc-Apt	ACV	0.01–100 ng/mL	0.010 ng/mL	AFB2, ZEN, OTA, FB1	Peanut	0.05 ng/mL	[93]
Apt1	GCE/THI-rGO/AuNPs/cDNA/MCH/Apt-Fc	ACV	0.05–20 ng/mL	0.016 ng/mL	FB1, AFB2, ZEN, OTA	Peanut	0.1 ng/mL	[94]
Apt1	GCE/AuNPs/β-CD/BSA/Fc-DNA	EIS	0.1 pg/mL–10 ng/mL	0.049 pg/mL	AFB2, OTA, FB1, ZEN, DON, SEB	Peanut oil	0.2 pg/mL	[95]
Apt1	GCE/Au@rGO/TDNs/BSA/Apt/cDNA/HPCS-Fc	DPV	0.01 pg/mL–100 µg/mL	0.033 pg/mL	AFB2, OTA, ATP, BSA	Wheat powder	1 ng/mL	[96]
Apt1	GCE/THI-rGO/CS/Apt/Fc-cDNA	ACV	0.001–100 ng/mL	0.330 pg/mL	AFB2, AFM1, OTA, ZEN	Peanut, peanut butter, peanut oil	0.1 ng/mL	[97]
Apt1	GCE/AQ-rGO/AuNPs/MB-HP/Apt-Fc	ACV	0.01 pg/mL–1 µg/mL	0.010 pg/mL	FB1, AFB2, ZEN, OTA	Peanut	1 pg/mL	[98]
COOH-Apt1-MB	SPCE/PT3C/HMDA/Apt-MB	DPV	2.5–30 ng/mL	1.600 pg/mL	OTA	Coffee	5 ng/mL	[99]
Apt1 v1 reversed	GCE/AuNPs/sDNA/MCH/Fc-Apt/Fc-aDNA	ACV	0.1–10000 pg/mL	0.012 pg/mL	FB1, ZEN, OTA	Corn powder	10 ng/mL	[100]
Apt1 v2	ITOE/AuNFs/cDNA-MB/MCH/Apt-Fc/AFB1/ExoI	DPV	0.1–1000 pg/mL	0.032 pg/mL	AFB2, FB1, ZEN, OTA, Glu, BSA, Na^+^, K^+^, Mg^2+^, Zn^2+^, Fe^3+^, Al^3+^	Peanut	0.1 ng/mL	[58]
SH-Apt1 v4	GE/Apt-MB/MCH	SWV	2.500 pg/mL–0.936 µg/mL	1.870 pg/mL	OTA, OTB, FB1, FB2, ZEN, AFG1, AFG2	White wine, milk, corn flour	2.500 pg/mL	[101]
SH-Apt1 v5	GE/Apt-MB/MCH	SWV	2.500 pg/mL–0.195 µg/mL	1.870 pg/mL	OTA, OTB, FB1, FB2, ZEN	Beer, grape juice, corn flour	2.500 pg/mL	[67]
NH_2_-(CH_2_)_6_-Apt3	GCE/α-Fe_2_O_3_-Fe_3_O_4_/CDs/Apt/MB	DPV	312.27 ng/mL–31.227 mg/mL	0.156 µg/mL	AFB2, AFM1, AFG1, AFG2, miscellaneous aspergillin (ST), OTA, FB1	Beer, rice, and peanut	1.560 ng/mL	[102]
Apt3 v2	GE/PTFE/sDNA-Fc/DNAzyme/Apt/Mn^2+^@MOF	DPV	0.1 pg/mL–1000 ng/mL	4.810 fg/mL	AFB2, AFG1, AFG2, AFM1, OTA, OTB, ZEN	Peanut oil	10 pg/mL	[103]
Competitive								
NH_2_-(CH_2_)_3_-Apt1	GCE/Ni-MOFs/AuNPs/MPA/Apt/BSA/cDNA/PBP	DPV	5 pg/mL–150 ng/mL	0.001 ng/mL	OTA, AFM1	Rice flour	0.750 ng/mL	[104]
BIO-Apt1	GE/cDNA/MCH/Apt-AFB1/SA/ CeO_2_/PorMOFs	DPV	0.03 pg/mL–3.12 ng/mL	9.360 fg/mL	AFB2, OTA, OTB	Peanut, cow milk	3.12 pg/mL	[105]
BIO-Apt1	GE/sDNA/MCH/HP1/HP2/SA-MBs/Ag^+^-DNAzyme/MB	DPV	1 pg/mL–50 ng/mL	0.416 pg/mL	T-2, OTA, ZEN, FB1, DON	Corn flour, buckwheat powder, walnut powder, white peony powder, wine	10 pg/mL	[69]
BIO-TEG-Apt1	SPCE/PANI-PAA/AFB1-BSA/Lys-Apt-BIO/SAP/1NP	DPV	0.1–10 ng/mL	0.086 ng/mL	AFG1	Corn flour	1 ng/mL	[50]
SH-Apt1	GCE/AuNPs/cDNA/MCH/Apt/(Exo I) AFB1-ssDNA-AuNPs-HRP	DPV	1 pg/mL–200 ng/mL	0.330 pg/mL	AFB2, AFG1, AFG2, AFM1, OTA, FB1	Peanut, corn	1 pg/mL	[57]
SH-Apt1	SPGE/Apt/cDNA/AFB1/MB	DPV	0.7–80 ng/mL	0.100 ng/mL	ZEN, OTA, AFM1, DON	Rat serum samples	10 ng/mL	[106]
Apt1	SiO_2_@PbS-Apt/MBs-CdTe/cDNA1/HindIII/MBs-cDNA2	SWV	5–50 ng/mL	4.500 pg/mL	FB1, OTA, AFB2, Glu, BSA, Ascorbic acid, K^+^, Fe^3+^	Peanut	0.500 ng/mL	[107]
Apt1 v1	GE/cDNA/MCH/Apt/HP1/HP2	DPV	0.01–100 pg/mL	2.840 fg/mL	AFB2, FB1, OTA, ZEN, DON	Corn, coix seed, polygala root	0.500 pg/mL	[108]
Apt1 v2-SH	GCE/AuNPs/Fc-Apt/MB-cDNA	SWV	0.3 pg/mL–3.12 ng/mL	0.037 pg/mL	AFG1, AFG2, AFM1, DON, ZEN	Peanut, corn, wheat	3.12 pg/mL	[109]
SH-Apt1 v3	GE/Apt-MB/MCH/cDNA	SWV	0.62 ng/mL–1.24 µg/mL	0.620 ng/mL	OTA, OTB, FB1, FB2, ZEN	Beer, white wine	0.620 ng/mL	[110]
SH-Apt1 v3	GE/cDNA/MCH/Apt-MB	SWV	0.62–156 ng/mL	0.620 ng/mL	OTA, OTB, FB1, FB2, ZEN	White wine	0.620 ng/mL	[111]
SH-(CH_2_)_6_-Apt1 v3	GE/Apt/Cys/cDNA/Fc-AuNPs	DPV	0.01–7.5 pg/mL	0.010 pg/mL	AFM1, AFB2, AFG1, OTA, ZEN	Beer	0.100 pg/mL	[112]
Apt4-SH	ITOE/AuNPs/Apt/MCH/cDNA-Au@Fe_3_O_4_	EIS, photothermal	10 pg/mL–300 ng/mL	0.005 ng/mL	OTA, OTB, FB1, AFM1	Peanut	0.100 ng/mL	[113]
Simultaneous								
AFB1: NH_2_-(CH_2_)_3_-Apt1OTA: NH_2_-C_6_H_12_-GATCGGGTGTGGGTGGCGTAAAGGGAGCATCGGACACGCCACCCACACA	GCE/AuNPs-CNDs/MPA/Apt(s)/BSA/Bioconj	DPV	0.01–100 ng/mL	AFB1: 5.200 pg/mL OTA: 4.300 pg/mL	AFM1	Corn flour	AFB1: 0.100 ng/mL OTA: 0.500 ng/mL	[114]
AFB1: Fc-Apt1 v1 OTA: GATCGGGTGTGGGTGGCGTAAAGGGAGCATCGGAC-MB	GE/AQ-hDNA/MCH/Fc-Apt1/MB-Apt2	ACV	AFB1: 10 pg/mL–3 ng/mL OTA: 30 pg/mL–10 ng/mL	AFB1: 4.300 pg/mL OTA: 13.300 pg/mL	AFB2, OTB, FB1, ZEN	Corn, wheat	AFB1: 0.100 ng/mL OTA: 0.100 ng/mL	[115]

Abbreviations: 1NP—1-naphtil phosphatase; ACV—alternating current voltammetry; aDNA—assistant DNA; AFB2—aflatoxin B2; AFG1—aflatoxin G1; AFG2—aflatoxin G2; AFM1—aflatoxin M1; Apt—aptamer; AQ—anthraquinone; AuNFs—gold nanoflowers; AuNPs—gold nanoparticles; ATP—adenosine triphosphate; AuNRs—gold nanorods; BDDE—boron-doped diamond electrode; BIO—biotin; Bioconj—bioconjugates; BSA—bovine serum albumin; CA—chronoamperometry; cDNA—complementary DNA; CDs—carbon dots; CNDs—carbon nanodots; CS—chitosan; Cu_2_O NCs—Cu_2_O nanocubes; CV—cyclic voltammetry; DON—deoxynivalenol; DPV—differential pulse voltammetry; dsDNA—double-stranded DNA; EIS—electrochemical impedance spectroscopy; Exo—exonuclease; FB1—fumonisin B1; FB2—fumonisin B2; Fc—ferrocene; GA—glutaraldehyde; GCE—glassy carbon electrode; GE—gold electrode; Glu—glucose; Gly—glycine; GNs—graphene nanosheets; GO—graphene oxide; HindIII—endonuclease; HMDA—hexamethylenediamine; hDNA—DNA hybridization chain reaction initiator; HP—hairpin DNA; HPCS—hollow porous carbon spheres; HRP—horseradish peroxidase; ITOE—indium tin oxide electrode; LDH NCs—layered double hydroxides nanocages; MB—methylene blue; MBs—magnetic beads; MCH—6-mercapto-1-hexanol; MOFs—metallic organic frameworks; MPA—3-mercaptopropionic acid; MIP—molecularly imprinted polymer; MWCNTs—multi-walled carbon nanotubes; NHS—N-hydroxysuccinimide; QDs—quantum dots; OTA—ochratoxin A; OTB—ochratoxin B; O-VMSF—3-glycidyloxypropyl trimethoxysilane-modified vertically ordered mesoporous silica film; P4VP—poly(4-vinyl pyridine); PAA—porous anodized aluminum; PANI—polyaniline; PANI-PAA—poly(aniline-anthranilic acid) copolymer; PBP—para-bisphenol; pDA—polydopamine; PDDA—poly(diallyl dimethylammonium chloride); PS—polystyrene; PTFE—polytetrafluoroethylene; PT3C—polythiophene-3 carboxylic; rGO—reduced graphene oxide; SA—streptavidin; SAP—streptavidin alkaline phosphatase; sDNA—substrate DNA; SEB—staphylococcal enterotoxin B; SGPGE—surface-graphenized pencil graphite electrode; SPCE—screen-printed carbon electrode; SPGE—screen-printed gold electrode; ssDNA—single-stranded DNA; SWV—square wave voltammetry; T-2—trichothecenes; TDNs—tetrahedral DNA nanostructures; TEG—triethylene glycol; TEMPO-COOH—4-carboxy-2,2,6,6-tetramethylpiperidine 1-oxyl free radical; THI—thionine; ZEN—zearalenone; ZIF-8—zeolitic imidazolate framework-8; *β*-CD—beta-cyclodextrin; *μ*PAD—microfluidic paper-based analytical device.

## Data Availability

Data sharing not applicable.
